# Simvastatin Enhances the Chondrogenesis But Not the Osteogenesis of Adipose-Derived Stem Cells in a Hyaluronan Microenvironment

**DOI:** 10.3390/biomedicines9050559

**Published:** 2021-05-17

**Authors:** Shun-Cheng Wu, Chih-Hsiang Chang, Ling-Hua Chang, Che-Wei Wu, Jhen-Wei Chen, Chung-Hwan Chen, Yi-Shan Lin, Je-Ken Chang, Mei-Ling Ho

**Affiliations:** 1 Regenerative Medicine and Cell Therapy Research Center, Kaohsiung Medical University, Kaohsiung 80701, Taiwan; shunchengwu@hotmail.com (S.-C.W.); linghua_chang@yahoo.com.tw (L.-H.C.); tkdiven@gmail.com (C.-W.W.); h90085663@gmail.com (J.-W.C.); hwan@kmu.edu.tw (C.-H.C.); sandylin27@hotmail.com (Y.-S.L.); 2 Orthopaedic Research Center, Kaohsiung Medical University, Kaohsiung 80701, Taiwan; milkpa@gmail.com; 3 Graduate Institute of Medicine, College of Medicine, Kaohsiung Medical University, Kaohsiung 80701, Taiwan; 4 Post-Baccalaureate Program in Nursing, Asia University, Taichung 41354, Taiwan; 5 Department of Orthopaedics, College of Medicine, Kaohsiung Medical University, Kaohsiung 80701, Taiwan; 6 Division of Adult Reconstruction Surgery, Department of Orthopedics, Kaohsiung Medical University Hospital, Kaohsiung Medical University, Kaohsiung 80701, Taiwan; 7 Department of Orthopedics, Kaohsiung Municipal Ta-Tung Hospital, Kaohsiung Medical University, Kaohsiung 80701, Taiwan; 8 Department of Physiology, College of Medicine, Kaohsiung Medical University, Kaohsiung 80701, Taiwan; 9 Department of Marine Biotechnology and Resources, National Sun Yat-sen University, Kaohsiung 80424, Taiwan; 10 Department of Medical Research, Kaohsiung Medical University Hospital, Kaohsiung Medical University, Kaohsiung 80701, Taiwan

**Keywords:** adipose-derived stem cells (ADSCs), simvastatin (SIM), hyaluronan microenvironment (HAM), chondrogenesis, osteogenesis

## Abstract

Directing adipose-derived stem cells (ADSCs) toward chondrogenesis is critical for ADSC-based articular cartilage regeneration. Simvastatin (SIM) was reported to promote both chondrogenic and osteogenic differentiation of ADSCs by upregulating bone morphogenetic protein-2 (BMP-2). We previously found that ADSC chondrogenesis is initiated and promoted in a hyaluronan (HA) microenvironment (HAM). Here, we further hypothesized that SIM augments HAM-induced chondrogenesis but not osteogenesis of ADSCs. ADSCs were treated with SIM in a HAM (SIM plus HAM) by HA-coated wells or HA-enriched fibrin (HA/Fibrin) hydrogel, and chondrogenic differentiation of ADSCs was evaluated. SIM plus HAM increased chondrogenesis more than HAM or SIM alone, including cell aggregation, chondrogenic gene expression (collagen type II and aggrecan) and cartilaginous tissue formation (collagen type II and sulfated glycosaminoglycan). In contrast, SIM-induced osteogenesis in ADSCs was reduced in SIM plus HAM, including mRNA expression of osteogenic genes, osteocalcin and alkaline phosphatase (ALP), ALP activity and mineralization. SIM plus HAM also showed the most effective increases in the mRNA expression of BMP-2 and transcription factors of SOX-9 and RUNX-2 in ADSCs, while these effects were reversed by CD44 blockade. HAM suppressed the levels of JNK, p-JNK, P38 and p-P38 in ADSCs, and SIM plus HAM also decreased SIM-induced phosphorylated JNK and p38 levels. In addition, SIM enhanced articular cartilage regeneration, as demonstrated by implantation of an ADSCs/HA/Fibrin construct in an ex vivo porcine articular chondral defect model. The results from this study indicate that SIM may be an enhancer of HAM-initiated MSC-based chondrogenesis and avoid osteogenesis.

## 1. Introduction

Damaged articular cartilage has a limited capacity for self-repair [1,2]. Cell-based tissue engineering may provide an alternative treatment method for cartilage defects [1,2], and chondrocytes or mesenchymal stem cells (MSCs) have been used to regenerate articular cartilage defects. Due to the poor proliferation capacity and de-differentiation of chondrocytes caused by in vitro expansion, using MSCs as the cell source is becoming an important approach for articular cartilage tissue engineering [3,4,5]. Adipose-derived stem cells (ADSCs) are thought to be a beneficial cell source for MSC-based articular cartilage tissue engineering due to their ease of harvest, high yield rate, and chondrogenic differentiation potential [6,7,8].

Chondrogenic induction of ADSCs is a key event in ADSC-based articular cartilage tissue engineering [1,9,10,11]. Among the chondrogenic growth factors, bone morphogenetic protein (BMP)-4, BMP-6, BMP-7, transforming growth factor (TGF)-βs, insulin-like growth factor (IGF)-1 and BMP-2 have been demonstrated to be the most effective [12,13,14]. BMP-2 is a member of the TGF-β superfamily and a potent regulator of cartilage growth and development [5,15,16]. BMP-2 has also been applied as a chondroinductive factor to promote MSC chondrogenesis [12,15,16,17,18]. However, BMP-2 also exerts osteogenic effects on MSCs [19,20,21,22]. Accordingly, the important concern in applying BMP-2 to stimulate MSC chondrogenesis for articular cartilage regeneration is to prevent undesired bone formation [23,24]. In addition to growth factors, simvastatin (SIM), a clinically used lipid-lowering agent, has been indicated to increase BMP-2 expression [25]. Accordingly, this study attempts to search for a strategy for applying SIM as an inducer and/or enhancer of chondrogenesis but not osteogenesis for articular cartilage tissue engineering.

Chondrogenesis is initiated by condensation (aggregation) of MSCs induced by SOX-9, a transcription factor for chondrogenesis, followed by the expression of chondrogenic genes (collagen type II and aggrecan) and subsequent formation of a hyaline cartilage extracellular matrix (ECM) consisting mainly of collagen type II and sulfated glycosaminoglycan (sGAG) [5,26]. Moreover, the niche, the natural microenvironment surrounding stem cells, regulates their survival, self-renewal and differentiation. Growth factors, cell–cell contacts and cell–matrix adhesions can be niche factors [27,28]. The ECM is an important niche during chondrogenesis [5]. Hyaluronan (HA) is a main ECM component in the mesenchyme during the early stage of chondrogenesis and is important in cartilage homeostasis [5,29,30]. We found that mimicking the HA microenvironment (HAM) of early-stage chondrogenesis can initiate and enhance ADSC chondrogenesis, increasing cell aggregation, chondrogenic gene (collagen type II & aggrecan) expression, and cartilaginous matrix formation [29,30,31,32]. These previous findings indicate that HA is a powerful contributor to creating the chondrogenic niche for MSCs. Accordingly, in this study, we hypothesized that SIM can augment HAM-induced chondrogenesis but not the osteogenesis of ADSCs and that this strategy can be used for articular cartilage regeneration.

## 2. Materials and Methods

### 2.1. Materials

Sodium HA with molecular weights (MWs) of 2000 kDa (FCH-200, MW = 1800~2200 kDa, average MW = 2000 kDa) was purchased from Kikkoman Co. (Tokyo, Japan). All chemicals were purchased from Sigma-Aldrich (St. Louis, MO, USA) unless otherwise specified.

### 2.2. Isolation and Culture of Human ADSCs

As in our previously described reports, ADSCs were isolated from subcutaneous adipose tissue obtained from human patients during orthopedic surgery [29,30,31,32]. Human adipose tissue was obtained from patients with approval from the ethics committee at Kaohsiung Medical University Hospital (KMUH-IRB-E(II)-20150193). After informed consent was obtained, subcutaneous adipose tissue was taken from the gluteal region of patients during orthopedic surgery. Three grams of human subcutaneous adipose tissue was minced with scissors and digested with 1 mg/mL type IA collagenase at 37 °C under 5% CO_2_ for 24 h. The digested tissue was centrifuged at 1000 rpm for 5 min, and the pellet was washed twice with PBS. The pellet was then resuspended in K-NAC medium and plated in a 100-mm culture dish. The K-NAC medium was used to facilitate ADSC isolation and expansion according to our previous reports [29,30,31,32,33]. The K-NAC medium was composed of keratinocyte-SFM basal medium (Gibco BRL, Rockville, MD, USA) supplemented with 25 mg of bovine pituitary extract (BPE) (Gibco BRL, Rockville, MD, USA), 2.5 µg of human recombinant epidermal growth factor (rEGF) (Gibco BRL, Rockville, MD, USA), 2 mM N-acetyl-L-cysteine, 0.2 mM L-ascorbic acid 2-phosphate sesquimagnesium salt, and 5% fetal bovine serum (FBS) [29,30,31,32,33]. Twenty-four hours after the initial plating, the K-NAC medium was refreshed, and the unattached cells were removed by washing twice with PBS. The medium was changed every 2 days thereafter, and the cells were allowed to grow to near confluence. After one week, a sufficient number of cells were produced for trypsinization and storage in liquid nitrogen or for subculture.

### 2.3. Cell Culture in HA-Coated Wells and Treatment with SIM

A coating of purified HA dissolved in distilled water was applied to 24-well plates (0 or 0.5 mg/cm^2^) for 48 h at 37 °C, after which the wells were washed twice with PBS [29,30,32]. SIM (Merck Sharp and Dohme Corp., Rahway, NJ, USA) was dissolved in dimethyl sulfoxide (DMSO) to prepare stock solutions. All reagents were diluted with basal medium (Dulbecco’s modified Eagle’s medium (DMEM) supplemented with 10% FBS, 1% nonessential amino acids and 100 U/mL penicillin/streptomycin (Gibco BRL)), or osteogenic differentiation medium (basal medium supplemented with L-ascorbic acid-2-phosphate (50 μM), β-glycerophosphate disodium (10 mM) and dexamethasone (0.1 μM) [34]), immediately before the initiation of treatment. SIM was used in these experiments at a concentration of 1 μM. The final concentration of DMSO in each treatment condition was <0.1% to reduce the influence of DMSO on the ADSCs. ADSCs were seeded at a density of 1 × 10^5^ cells/500 μL of medium and treated with SIM. ADSCs were divided into four groups as follows: control group—ADSCs were cultured in wells and left untreated; SIM group—ADSCs were cultured in wells and treated with SIM; HAM group—ADSCs were cultured in HA-coated wells; and SIM+HAM group—ADSCs were cultured in HA-coated wells and treated with SIM. The medium was changed every 2 days. At every indicated time interval, ADSCs were collected for further experimental analysis.

### 2.4. RNA Isolation and Quantitative Real-Time Polymerase Chain Reaction (Real-Time PCR)

At the indicated time points, ADSCs from each experimental group cultured in the wells were collected. TOOLSmart RNA Extractor (TOOLS, Taipei, Taiwan) was used to extract total RNA from these ADSCs. The RNA quality was confirmed by determining the A260/A280 absorbance ratio using a Thermo Scientific NanoDropTM 1000 spectrophotometer (Thermo Fisher Scientific, Waltham, MA, USA). An A260/A280 absorbance ratio in the range of 1.8–2.0 was considered to indicate the absence of DNA contamination according to the manufacturer’s instructions. Total RNA (0.5–1 μg per 20 μL reaction volume) was reverse-transcribed to cDNA using the TOOLS Easy Fast RT Kit (TOOLS, Taipei, Taiwan). Real-time PCR was performed using TOOLS 2X SYBR qPCR Mix (TOOLS, Taipei, Taiwan) in a quantitative real-time PCR detection system (Bio-Rad Laboratories Inc., Hercules, CA, USA). cDNA samples (2 µL samples in a total volume of 25 µL per reaction) were analyzed for the genes of interest. Previously published primer sequences were used to detect BMP-2, SOX-9, collagen type II, aggrecan, runt-related transcription factor 2 (RUNX-2), osteocalcin (OC), alkaline phosphatase (ALP) and glyceraldehyde-3-phosphate-dehydrogenase (GAPDH) [29,30,31,32,35,36,37]. All primer sequences used in this study are listed in Table 1. After real-time PCR was conducted, a dissociation (melting) curve was generated to determine the specificity of the reaction. The relative mRNA expression level of each target gene was calculated from the threshold cycle (Ct) value of each PCR product and normalized to the expression of GAPDH using the comparative Ct method [38]. For each gene of interest, readings were collected in each experimental group at every indicated time point.

### 2.5. Western Blot Analysis

At each indicated time point, cells from each group were washed twice with ice-cold PBS supplemented with 1 mM sodium vanadate and lysed in modified radioimmunoprecipitation assay (RIPA) buffer (150 mM NaCl, 1 mM EGTA, 50 mM Tris (pH 7.4), 10% glycerol, 1% Triton X-100, 1% sodium deoxycholate, 0.1% SDS) containing a protease inhibitor cocktail (Complete Protease Inhibitor Cocktail Tablets; Roche Diagnostics Ltd., Taipei, Taiwan) and 1 mM sodium vanadate. The lysates were cleared by centrifugation at 14,000 rpm for 15 min at 4 °C. The proteins were quantitated using the BCA protein assay. The protein expression levels were analyzed by Western blotting using antibodies against BMP-2 (catalog number: bs-1012R; Bioss, Beijing, China), anti-p38 (catalog number: 14064-1-AP; Proteintech, Rosemont, IL, USA), anti-p-p38 (catalog number: AP0526; ABclonal, Wuhan, China), anti-JNK (catalog number: ARG51218; Arigo, Hsinchu, Taiwan), anti-p-JNK (catalog number: ARG51807; Arigo, Hsinchu, Taiwan) and β-actin (catalog number: A5441), and immunoreactions were visualized using an enhanced chemiluminescence (ECL) system (Amersham, UK).

### 2.6. Alcian Blue Staining for Detection of sGAG Deposition

To assess sGAG deposition, ADSCs from each group were subjected to Alcian blue staining. Before Alcian blue staining, ADSCs were washed at least three times with PBS to remove HA residue remaining on the tissue culture plate and were then fixed overnight with 4% paraformaldehyde in PBS. Staining to assess sGAG deposition was performed by applying a solution of 0.1% Alcian blue 8 GX in 0.1 M HCl to the cells for 2 h at room temperature.

### 2.7. Dimethylmethylene Blue (DMMB) Assay for Quantification of sGAG Deposition

The DMMB assay was used to quantify sGAG deposition by ADSCs. DMMB reacts with the sulfate group of the GAG chain and will not react with nonsulfated GAGs such as HA [39]. At each indicated time point, the DNA content and sGAG deposition of each sample were quantified spectrofluorometrically using Hoechst 33,258 dye and DMMB, respectively [34,36,40,41]. A standard curve for the DMMB assay was generated using an aqueous solution of chondroitin sulfate C (Sigma-Aldrich, St. Louis, MO, USA) with concentrations ranging from 0 to 25 μg/μL.

### 2.8. Enzyme-Linked Immunosorbent Assay (ELISA) for Quantification of Collagen Type II Synthesis

At each indicated time point, the collagen type II content of each sample was measured using a Type II Collagen Detection Kit (Chondrex, Redmond, WA, USA) [42]. The DNA content was measured for normalization using a Quant-iT Picogreen dsDNA Assay Kit (Invitrogen, Waltham, MA, USA). The standard curve for collagen type II was generated using different concentrations of collagen type II [42].

### 2.9. Alizarin Red S Staining and Quantification

Calcium deposition in a calcified matrix was assessed with Alizarin red S staining [43,44]. At each indicated time point, the ADSCs were fixed with 0.05% (*v*/*v*) glutaraldehyde at room temperature for 10 min and were then washed with distilled water. Next, the fixed ADSCs were incubated with Alizarin red S (1% in distilled water, pH 4.2) for 5 min and then extensively washed with distilled water. The fixed and stained plates were then air dried at room temperature. Staining of calcium mineral deposits was imaged. To determine the amount of calcium deposition, ADSC-bound Alizarin red S was dissolved in 10% acetic acid and quantified spectrophotometrically at 415 nm.

### 2.10. Alkaline Phosphatase (ALP) Activity

An ALP kit (catalog number: E-BC-K091-S; Elabscience, Wuhan, China) was used to detect ALP activity according to the manufacturer’s instructions. At each indicated time point, ADSCs were washed with PBS twice and then centrifuged at 1000× *g* for 10 min. The supernatant was discarded, and the cell sediment was retained. Next, PBS was added at a ratio of cell number (106): PBS (μL) = 1:500 and sonicated in an ice water bath for 15 min. The sonicated cell lysate was centrifuged at 10,000× *g* for 10 min, and the supernatant was collected and preserved on ice for detection. To determine the ALP activity, the reaction mixture was added to each sample, incubated for 30 min and then quantified spectrophotometrically at 520 nm.

### 2.11. Receptor Binding Inhibition Assay

The HA receptor binding inhibition assay was performed by treating ADSCs with an anti-CD44 blocking antibody. The anti-CD44 antibody (Clone IM7, eBioscience; catalog number: 14-0441-82) was used to block the binding of HA to the CD44 receptor [40,41]. ADSCs were pretreated with 10 μg/mL anti-CD44 antibody for 2 h at 37 °C under 5% CO_2_ [40,41], then resuspended in 1 mL of basal medium containing 10 μg/mL anti-CD44 antibody and seeded in HA-coated wells. The basal medium containing 10 μg/mL anti-CD44 antibody was changed every 2 days. At each indicated time interval, cells were collected for further experimental analysis.

### 2.12. Encapsulation of ADSCs in the 3D HA-Enriched Fibrin (HA/Fibrin) Hydrogel and Treatment with SIM

The 3D HA/Fibrin hydrogels were synthesized according to a method described in our previously published research [29,31]. In brief, fibrinogen was first isolated from minipigs by a previously described ammonium sulfate method [45], dissolved in PBS as a 100 mg/mL fibrin solution, and stored at −80 °C. To encapsulate ADSCs in the Fibrin and HA/Fibrin hydrogels, ADSCs were premixed in PBS or in 1% HA solution. For hydrogel formation, every 30 μL of ADSCs (1 × 10^6^ cells) suspended in PBS or HA was mixed with 120 μL of fibrin solution (100 mg/mL) and then placed in a Teflon mold 5.5 mm in depth and 5.5 mm in diameter. A 40 μL volume of bovine thrombin (300 U/mL) in 40 mM CaCl_2_ was added to the mold and mixed. The mixtures were incubated at room temperature for 15 min to form hydrogels. After hydrogel formation, the 3D hydrogel constructs containing ADSCs with PBS (ADSC/Fibrin) or ADSCs with HA/Fibrin (ADSC/HA/Fibrin) were transferred to a 24-well plate and cultured with 1 mL of chondrogenic medium (basal medium supplemented with 6.25 μg/mL insulin (catalog number: I1882), 10 ng/mL TGF-β1 (catalog number: T1654) and 50 μM ascorbic acid-2-phosphate (catalog number: A8960) [32]) treated w/o 1 µM SIM. The 3D hydrogel constructs were divided into four groups as follows: Control group—3D ADSC/Fibrin constructs were cultured in wells and left untreated; SIM group—3D ADSC/Fibrin constructs were cultured in wells and treated with SIM; HAM group—3D ADSC/HA/Fibrin constructs were cultured in wells and left untreated; and SIM+HAM group—ADSC/HA/Fibrin constructs were cultured in wells and treated with SIM. The medium was changed every 2 days. At every indicated time interval, the constructs were collected for further experimental analysis.

### 2.13. Chondral Defect Repair Ability of SIM and the 3D HA/Fibrin Hydrogel in ADSCs

We previously established an ex vivo model of chondral defects in osteochondral core explants [31]. This model was used to test the chondral defect repair ability of SIM and 3D HA/Fibrin hydrogels in ADSCs. Briefly, porcine knee joints were purchased from a slaughterhouse immediately after the pig was slaughtered. The femoral condyle was exposed under sterile conditions, and chondral defects 2 mm in diameter were created by drilling into the cartilage layer of the condyle. Then, the osteochondral cores (diameter: 6 mm) with chondral defects (diameter: 2 mm) at the center were detached from the femoral condyle using a trephine bur. After detachment, the osteochondral core explants were sterilized by washing with 5% gentamycin in PBS (*v*/*v*) for 30 min and were then washed 3 times with sterile PBS to minimize the amount of bone marrow adhering to the osteochondral cores. The osteochondral cores were then transferred to a 24-well plate for culture. First, the osteochondral cores were attached to the bottom of the wells using 100 μL of Histoacryl^®^ Blue. After attachment, the chondral defects were filled with hydrogel constructs of ADSC/Fibrin (1 × 10^6^ cells) or ADSC/HA/Fibrin (1 × 10^6^ cells). After the hydrogels were formed within the defect sites in the osteochondral cores, 2 mL of basal medium was added to each well to cover the osteochondral cores. The ex vivo study was performed with five groups as follows: empty—chondral defects without implantation of any substance; HAM—chondral defects implanted with HA/Fibrin; ADSC—chondral defects implanted with ADSC/Fibrin; HAM/ADSC—chondral defects implanted with ADSC/HA/Fibrin; and SIM/HAM/ADSC—chondral defects implanted with ADSC/HA/Fibrin and supplemented with SIM (1 μM) treatment. The basal medium was refreshed every 2 days for 4 weeks. At the indicated time points, the osteochondral cores in each group were harvested for further analysis.

### 2.14. Histological and Quantitative Analyses

Histological analysis of the osteochondral cores was performed based on our previously reported method [31]. After 4 weeks of cultivation, the osteochondral cores in each group were harvested. Samples were fixed with 10% formalin in PBS for 24 h and were then decalcified with 10% formic acid in PBS for 7 days. All osteochondral cores were then embedded in paraffin and sectioned longitudinally at a thickness of 5 µm. Safranin O and fast green staining were used to evaluate the sGAG content. Neoformation of cartilaginous tissue was used to compare the chondral defect repair associated with the implanted constructs in each group [31].

To analyze the neoformation of cartilaginous tissue in each group, samples were first sectioned and stained with safranin O and fast green. Then, neoformation of cartilaginous tissue at the defect site was quantitated using Image-Pro Plus 5.0 software (Media Cybernetics, Silver Spring, MD, USA). The chondral defect sites in each group are circled with a green line. The ratio of the matrix-stained area to the defect site area was calculated. Neoformation of cartilaginous tissue was quantitated using the following formula: percentage of cartilaginous tissue neoformation = matrix-stained area within the chondral defect site area/total chondral defect site area [31].

### 2.15. Statistical Analysis

The data are expressed as the means ± standard errors of the mean (SEMs) of the combined results of the experimental replicates. Statistical significance was evaluated using one-way analysis of variance (ANOVA), and multiple comparisons were performed using Scheffe’s method. Values of *p* < 0.05 were considered to indicate significant differences.

## 3. Results

### 3.1. SIM Plus HAM Augments the Chondrogenesis of ADSCs

**Figure 1:**
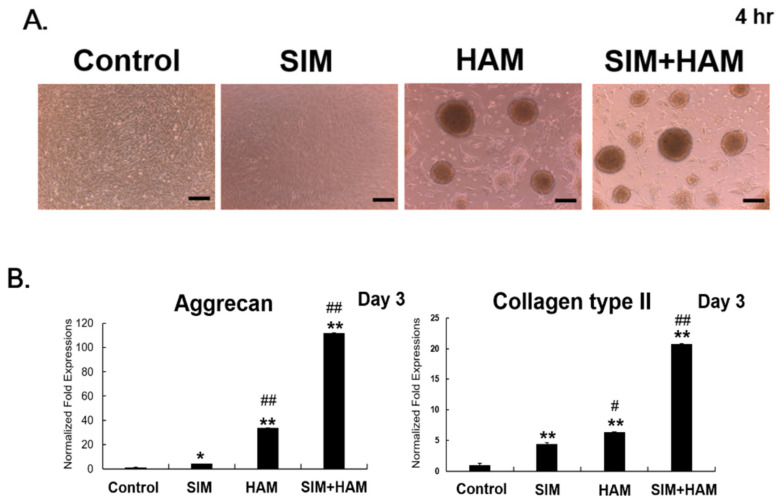

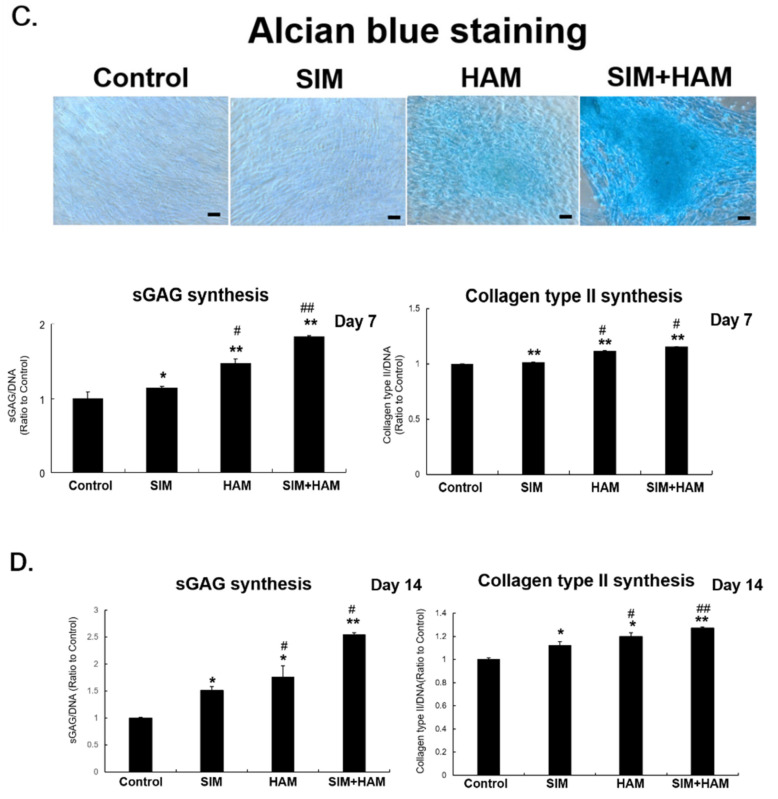
SIM plus HAM enhances the chondrogenesis of ADSCs. ADSCs were treated with SIM and cultured in wells with/without an HA coating, and the chondrogenesis of ADSCs was analyzed. (**A**) Cell aggregation of ADSCs in the control, SIM, HAM and SIM+HAM groups at 4 h (scale bar = 200 μm). (**B**) The mRNA expression levels of chondrogenic genes (aggrecan and collagen type II) in the control, SIM, HAM and SIM+HAM groups on day 3. The gene expression levels are expressed relative to those in the control group, which are defined as 1. (**C**) Alcian blue staining of sGAG in the control, SIM, HAM and SIM+HAM groups on day 7. Blue: Alcian blue staining (scale bars = 200 μm). sGAG synthesis and collagen type II synthesis by ADSCs were quantified with a DMMB assay and an ELISA kit, respectively. The abundance of synthesized sGAG or collagen type II normalized to the total DNA concentration in each group is expressed as the sGAG/DNA or collagen type II/DNA ratios. The sGAG/DNA and collagen type II/DNA ratios are expressed relative to that in the control group on day 7, which is defined as 1. (**D**) ADSCs were embedded in a 3D HA-enriched fibrin hydrogel (HA/Fibrin) and treated with SIM in chondrogenic medium for 14 days. sGAG synthesis and collagen type II synthesis by ADSCs were quantified with a DMMB assay and an ELISA kit, respectively. The abundance of synthesized sGAG or collagen type II normalized to the total DNA concentration in each group is expressed as the sGAG/DNA or collagen type II/DNA ratios. The sGAG/DNA and collagen type II/DNA ratios are expressed relative to that in the control group on day 14, which is defined as 1. The values presented are the means ± SEMs (*n* = 6). (*) and (**) indicate *p* < 0.05 and *p* < 0.01, respectively, compared with the control group. (#) and (##) indicate *p* < 0.05 and *p* < 0.01, respectively, compared with the SIM group.

To determine whether SIM plus HAM enhances the chondrogenesis of ADSCs, ADSC chondrogenesis was evaluated by assessing cell aggregation, the mRNA expression levels of chondrogenic genes (collagen type II and aggrecan) and the deposition of sGAG by ADSCs in the four groups. Cell aggregates were not obviously found in either the Control or SIM group but were observed in the HAM group (Figure 1A), and more pronounced cell aggregation was found in the SIM+HAM group than in the HAM group (Figure 1A). In addition, the mRNA expression of chondrogenic genes (aggrecan and collagen type II) was significantly increased in both the SIM and HAM groups compared with the control group. The SIM+HAM group showed the most pronounced increase in the expression levels of chondrogenic genes among the four groups (Figure 1B). Alcian blue staining, a DMMB assay, and collagen type II ELISA were used to examine sGAG deposition and collagen type II synthesis by ADSCs in the four groups. The sGAG deposition and collagen type II synthesis results showed an effect consistent with the mRNA expression levels of chondrogenic genes (Figure 1C). To further determine the effect of SIM in a HAM on cartilaginous tissue formation from ADSCs, the formation of cartilaginous tissue from 3D ADSC/HA/Fibrin constructs after SIM treatment was evaluated on day 14 after culture in chondrogenic medium. The results of sGAG deposition and collagen type II synthesis under 3D culture supported the previous results from 2D culture. The results confirmed that the constructs in the SIM+HAM group showed higher levels of sGAG production and collagen type II synthesis than the other 3 groups on day 14 (Figure 1D). Collectively, these results show that SIM plus HAM showed the best enhancing effect on the chondrogenesis of ADSCs (Figure 1).

### 3.2. SIM Plus HAM Reduces the Osteogenesis of ADSCs

**Figure 2:**
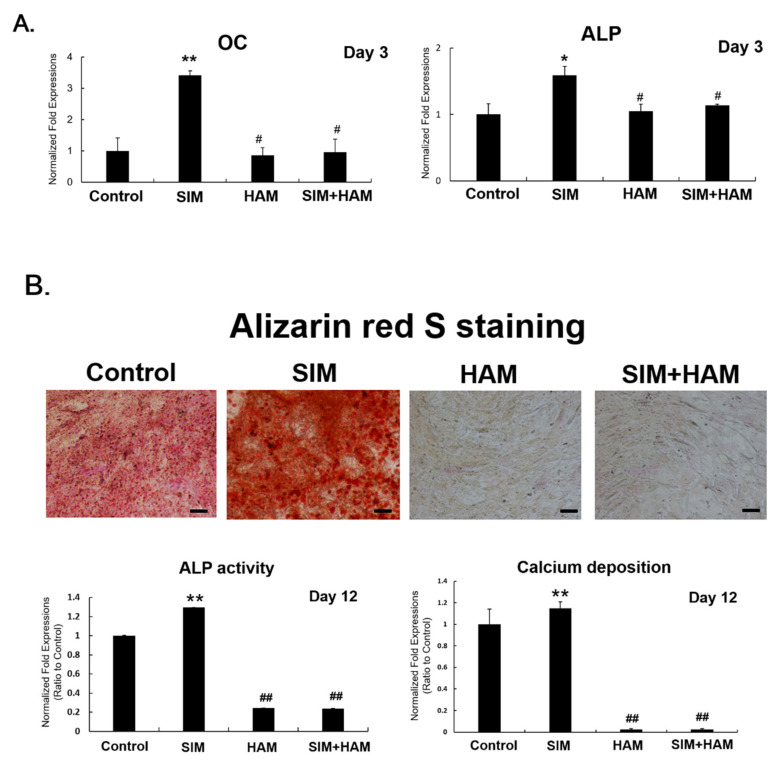
SIM plus HAM reduces the osteogenesis of ADSCs. ADSCs were treated with SIM and cultured in wells with/without an HA coating in basal medium, and the osteogenesis of ADSCs was analyzed. (**A**) The mRNA expression levels of osteogenic genes (osteocalcin; OC and alkaline phosphatase; ALP) in the control, SIM, HAM and SIM+HAM groups on day 3. The gene expression levels are expressed relative to those in the control group, which are defined as 1. (**B**) ADSCs were treated with SIM and cultured in wells with/without an HA coating in osteogenic medium, and the mineralization of ADSCs was analyzed. ALP activity and Alizarin red S staining of calcium deposition were performed on day 12. Red: Alizarin red S staining. The abundance of ALP activity and calcium deposition are expressed relative to that in the control group on day 12, which is defined as 1. The values presented are the means ± SEMs (*n* = 6). (*) and (**) indicate *p* < 0.05 and *p* < 0.01, respectively, compared with the control group. (#) and (##) indicate *p* < 0.05 and *p* < 0.01, respectively, compared with the SIM group.

The effect of SIM plus HAM on the osteogenesis of ADSCs was also examined. ADSC osteogenesis was evaluated by assessing the mRNA expression levels of osteogenic genes (OC and ALP) as well as the deposition of calcium and ALP activity by ADSCs. SIM plus HAM reduced the osteogenic effect of SIM on ADSCs. The mRNA expression levels of osteogenic genes (OC and ALP) were significantly increased in the SIM group compared to the control group (Figure 2A). However, significant decreases in the mRNA expression levels of osteogenic genes were found in the HAM and SIM+HAM groups compared with the SIM group (Figure 2A). No significant difference was found among the control, HAM and SIM+HAM groups. Moreover, decreased osteogenesis of ADSCs in the HAM and SIM+HAM groups were observed in the ALP activity and Alizarin red staining and quantification assays (Figure 2B). The results showed that SIM-induced ALP activity and calcium deposition on ADSCs were reduced in the HAM and SIM plus HAM groups (Figure 2B). Overall, the results show that SIM plus HAM increases chondrogenesis but not osteogenesis of ADSCs in vitro.

### 3.3. SIM Plus HAM Enhances the mRNA Expression of Transcription Factors for ADSC Chondrogenesis and Osteogenesis, But This Effect Is Reversed by HA-CD44 Blockade

**Figure 3:**
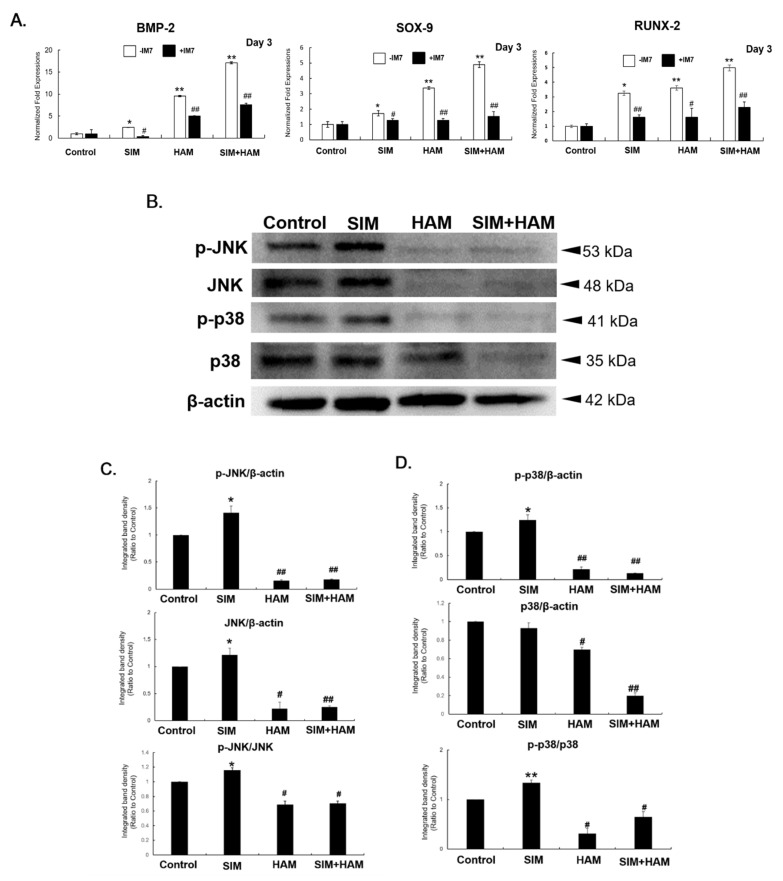
SIM plus HAM enhances the mRNA expression of transcription factors for ADSC chondrogenesis and osteogenesis. ADSCs were pretreated with IM7 (+IM7) or without IM7 (−IM7) prior to SIM treatment and cultivated in wells with/without an HA coating, and the mRNA expression levels of (**A**) BMP-2, SOX-9 and RUNX-2 in the control, SIM, HAM and SIM+HAM groups on day 3 were analyzed. The mRNA levels are expressed relative to those in the control group, which are defined as 1. The values presented are the means ± SEMs (*n* = 6). (*) and (**) indicate *p* < 0.05 and *p* < 0.01, respectively, compared with the control group. (#) and (##) indicate *p* < 0.05 and *p* < 0.01, respectively, and represent the comparison between +IM7 and -IM7 in each group. (**B**) p38 and JNK signaling were analyzed by Western blot. Representative Western blot photographs were shown. (**C**) The p-JNK/β-actin, JNK/β-actin and p-JNK/JNK ratios and (**D**) p-p38/β-actin, p38/β-actin and p-p38/p38 ratios are expressed relative to that in the control group on day 3, which is defined as 1. The values presented are the means ± SEMs (*n* = 6). (*) and (**) indicate *p* < 0.05 and *p* < 0.01, respectively, compared with the control group. (#) and (##) indicate *p* < 0.05 and *p* < 0.01, respectively, compared with the SIM group.

To determine whether SIM plus HAM also induces the expression of transcription factors for chondrogenesis and osteogenesis in ADSCs, the mRNA expression levels of BMP-2, SOX-9 and RUNX-2 in ADSCs in the four groups were measured by real-time PCR. Compared to ADSCs in the control group, ADSCs in the SIM and HAM groups exhibited increased mRNA expression levels of BMP-2, SOX-9 and RUNX-2 (Figure 3A). Moreover, even more pronounced increases in BMP-2, SOX-9 and RUNX-2 expression levels were found in ADSCs in the SIM+HAM group than in ADSCs in the SIM group (Figure 3A). On the other hand, inhibiting the interaction between HA and CD44 (HA-CD44 interaction) with IM7 significantly reduced the HAM-induced increase in SIM-induced BMP-2, SOX-9 and RUNX-2 expression in ADSCs (Figure 3A). These results show that SIM plus HAM enhances the expression of not only BMP-2 but also transcription factors for both chondrogenesis and osteogenesis in ADSCs. On the other hand, the HA-CD44 interaction is critical for the SIM plus HAM-mediated enhancement of BMP-2, SOX-9 and RUNX-2 expression in ADSCs.

### 3.4. HAM and SIM Plus HAM Inhibit Mitogen-Activated Protein Kinase (MAPK) Activation

To determine whether mitogen-activated protein kinase (MAPK) activation contributed to reducing the osteogenesis of ADSCs, we assessed the expression and phosphorylated forms of JNK and p38 (Figure 3B). p-JNK/JNK and p-p38/p38 were increased in ADSCs in the SIM group compared with the control group. However, the levels of JNK, p-JNK, P38 and p-P38 were suppressed in the HAM group, and p-JNK/JNK and p-p38/p38 were decreased in the HAM and SIM+HAM groups (Figure 3C,D).

### 3.5. SIM Plus 3D HAM Increases Neoformation of Cartilaginous Tissue from ADSCs at the Articular Cartilage Defect Site

**Figure 4:**
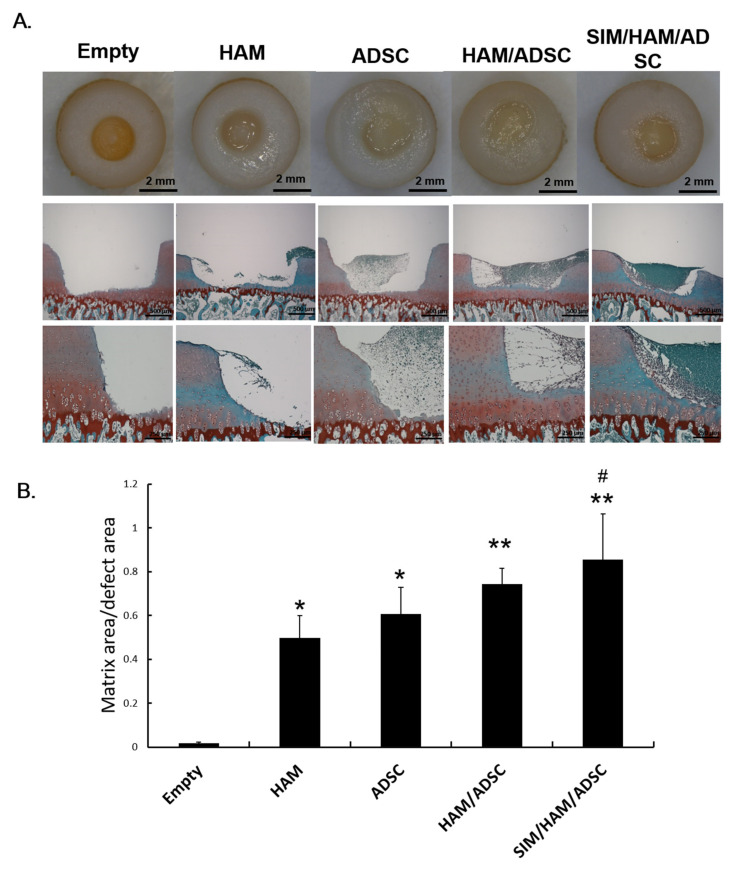
SIM plus 3D HAM increases the neoformation of cartilaginous tissue by ADSCs at the articular cartilage defect site. (**A**) Histological images of each group after safranin O and fast green staining. Representative micrographs of the empty, HAM, ADSC, HAM/ADSC and SIM/HAM/ADSC groups are shown. sGAG was stained red, and green indicates the counterstain. The original magnification was 40× (scale bar = 1000 μm) or 100× (scale bar = 500 μm). (**B**) Quantitative analysis of the percentage of neocartilaginous tissue formed at the defect site. The values indicate the ratio of neocartilaginous tissue formation to the defect area after 4 weeks of culture. The values are the means ± SEMs (*n* = 5). (*) and (**) indicate *p* < 0.05 and *p* < 0.01, respectively, compared with the empty group. (#) indicates *p* < 0.05 compared with the HAM/ADSC group.

After 4 weeks of culture, neoformation of cartilaginous tissue at the defect site of each group was examined (Figure 4A). The gross morphology of the osteochondral core in each group showed that the defect sites in the construct-implanted groups were filled with more repaired tissue than those in the empty group. In the HAM group, only a small amount of undegraded implanted hydrogel remained at the defect sites. In the ADSC group, a limited amount of cartilaginous tissue neoformation was found at the defect site (Figure 4A). In the HAM/ADSC and SIM/HAM/ADSC groups, the defects were almost completely filled with repaired tissue (Figure 4A).

The samples were further sectioned and stained with safranin O and fast green, and staining was quantified in all groups. Quantitative analysis of cartilaginous tissue neoformation demonstrated significant increases in the HAM, ADSC, HAM/ADSC and SIM/HAM/ADSC groups compared to the empty group (Figure 4B). Moreover, the SIM/HAM/ADSC group showed significantly higher matrix content at the defect site than the other groups (Figure 4B). This result indicated that SIM plus 3D HAM increases the neoformation of cartilaginous tissue from ADSCs at the articular cartilage defect site.

### 3.6. The optimal Timing at which SIM Plus HAM Enhances BMP-2 Expression in ADSCs Is Day 3

**Figure 5:**
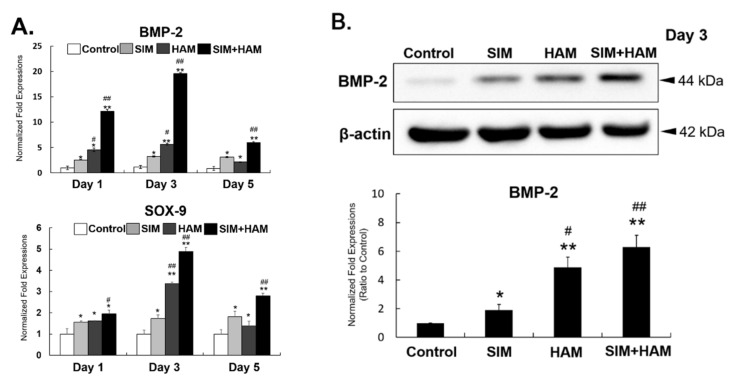
The optimal timing at which SIM plus HAM enhances BMP-2 expression in ADSCs is day 3. ADSCs were treated with SIM and cultured in wells with/without HA coating. BMP-2 and SOX-9 expressions in ADSCs were analyzed from days 1 to 5. (**A**) mRNA expression levels of BMP-2 and SOX-9 in the control, SIM, HAM and SIM+HAM groups from days 1 to 5. The gene expression levels are relative to those in the control group, which are defined as 1. (**B**) The protein expression levels of BMP-2 and β-actin in the control, SIM, HAM and SIM+HAM groups on day 3. The BMP-2/β-actin expression ratio of each group is relative to that of the control group, which is defined as 1. The values presented are the means ± SEMs (*n* = 4). (*) and (**) indicate *p* < 0.05 and *p* < 0.01, respectively, compared with the control group. (#) and (##) indicate *p* < 0.05 and *p* < 0.01, respectively, compared with the SIM group.

To determine the optimal time effect of SIM plus HAM on the mRNA or protein levels of BMP-2 and SOX-9 in ADSCs, BMP-2 and SOX-9 expression was measured in the four groups of ADSCs by real-time PCR and Western blot analyses. Compared to ADSCs in the control group, ADSCs in both the SIM and HAM groups exhibited increased BMP-2 and SOX-9 mRNA expressions from days 1 to 5 (Figure 5A). An even more pronounced increase in BMP-2 and SOX-9 mRNA expressions was found in ADSCs in the SIM+HAM group than in ADSCs in the SIM and HAM groups from days 1 to 5. Moreover, the most obvious effect of SIM plus HAM on BMP-2 mRNA expression in ADSCs was found on day 3 (Figure 5A). A similar effect on BMP-2 protein expression was also observed in the four groups of ADSCs on day 3 (Figure 5B). These results show that SIM plus HAM further enhances SIM-induced BMP-2 expression in ADSCs, and the optimal effect was observed on day 3 (Figure 5A,B).

## 4. Discussion

Effective enhancement of ADSC chondrogenesis and hyaline cartilage formation remains a clinically unmet need in ADSC-based articular cartilage tissue engineering [11,46]. SIM is a clinically used lipid-lowering agent that has been indicated to be a potent chondroinductive factor due to its induction of BMP-2 expression in MSCs [47,48]. However, BMP-2 enables the induction of both the chondrogenesis and osteogenesis of MSCs [19,20,21,22,49]. We proposed that SIM may be an enhancer to augment HAM-initiated chondrogenic but not osteogenic effects on MSCs. In this study, we demonstrated that in HAM, SIM augments the promotive effect of HA on chondrogenesis but does not affect HA-decreased osteogenesis of ADSCs. Moreover, the addition of SIM further augments ADSC/HA/Fibrin 3D construct-induced articular cartilage regeneration. This finding indicates that SIM can potentiate HA niche-enhanced ADSC-based articular cartilage regeneration.

In this study, we first found that SIM plus HAM has a synergistic effect on enhancing the chondrogenesis of ADSCs rather than SIM or HAM alone, as evidenced by increased cell aggregation, the mRNA expression levels of aggrecan and collagen type II, and cartilaginous matrix formation (sGAG deposition and collagen type II synthesis) (Figure 1A–D). More importantly, we also found the synergistic effect of SIM plus HAM on increasing BMP-2 expression in ADSCs (Figure 5). In MSCs, SIM has been indicated to increase the expression of BMP-2, a potent regulator of both chondrogenesis and osteogenesis [5,49]. BMP-2 has also been shown to promote cell aggregation and SOX-9 expression during chondrogenesis [5,50], and SOX-9 can further regulate collagen type II and aggrecan expression in MSCs [51,52]. We suggest that the synergistic enhancing effect of SIM plus HAM on BMP-2 expression in ADSCs may subsequently promote a more pronounced effect on ADSC chondrogenesis than SIM or HAM alone.

In this study, we found that cell aggregations were observed in the HAM group, and more pronounced cell aggregation was found in the SIM+HAM group than in the HAM group (Figure 1A). Cartilage formation begins in the mesenchyme by the condensation and differentiation of MSCs in pre-chondrocytes [53]. The cell aggregation of MSCs into pre-cartilage condensations is one of the earliest events when chondrogenesis is triggered [54]. HA is essential for cell-to-cell cross bridging for cell aggregation prior to pre-cartilaginous condensation [55]. Our previous studies also indicate that the HAM initiates cell aggregation and promotes chondrogenesis of ADSCs [29,30,31,32]. On the other hand, SIM has been indicated to increase BMP-2 expression [27]. BMP-2 plays a major role in the condensation phase of MSCs during chondrogenesis [5]. BMP-2 has been shown to promote cell aggregation during chondrogenesis [5,50]. We also found that more enhanced BMP-2 expression of ADSCs in SIM+HAM group than in the HAM group (Figure 5). Collectively, we suggest that the more pronounced cell aggregation that was found in the SIM+HAM group than in the HAM group may due to the initiation of cell aggregation by HAM combined with the SIM-induced BMP-2 expression in ADSCs.

During early stage of chondrogenesis process, aggrecan is an important chondrogenic marker gene [5]. MSCs undergo cell aggregation (condensation) followed by the expression of chondrogenic genes (collagen type II and aggrecan) during early stage of chondrogenesis process [5,26]. The mRNA expressions of collagen type II and aggrecan indicate that the MSCs are committed to chondrogenesis [5,26]. In the later stage of chondrogenesis, MSCs chondro-differentiate into chondrocytes and start to synthesize the hyaline cartilage [5]. Hyaline cartilage is mainly composed of collagen type II and sulphated glycosaminoglycan (sGAG) [2,29,31,32]. sGAG contributes to the mechanical stability of hyaline cartilage via forming an interwoven meshwork with collagen type II [56]. Aggrecan is only a core protein linking covalently with sGAG to maintain the chondrocyte phenotype [57]. Therefore, we detect the mRNA expression of aggrecan only on day 3, and the sGAG deposition was detected on day 7 under 2D culture (Figure 1). In the 3D HA-enriched fibrin hydrogel model, we also detect the sGAG and collagen type II on day 14 (Figure 1).

We have previously shown that 2D HAM initiated and promoted on chondrogenesis of ADSCs [30,32,58]. We also found that neoformation of cartilaginous tissue (Collagen type II synthesis or sGAG deposition) by ADSCs under a 2D HAM was detectable from day 5 to 10 [30,32,58]. In a 3D culture, we also have showed that the neoformation of cartilaginous tissue of ADSCs was detectable from day 10 to 21 cultured in a HA/fibrin hydrogel or a pellet culture [31,35,58]. Therefore, we detect the neoformation of cartilaginous tissue (Collagen type II synthesis or sGAG deposition) by ADSCs under a 2D HAM on day 7. In the 3D HA-enriched fibrin hydrogel model, we detect the neoformation of cartilaginous tissue (Collagen type II synthesis or sGAG deposition) on day 14.

However, BMP-2 is also a potent regulator of osteogenesis; therefore, the effect of SIM plus HAM on the osteogenesis of ADSCs was further evaluated. MSC osteogenesis is characterized by the expression of osteogenic genes (BMP2, RUNX-2, etc.), ECM markers (ALP and OC), subsequent ALP activity and final calcium deposition in the ECM [58,59]. BMP-2 promotes RUNX-2 expression, which is the principal transcriptional regulator of osteogenesis and is required for the expression of ALP and OC [60,61,62]. We found that although SIM alone increased ADSC osteogenesis, including ALP and OC gene expression and ALP activity and mineralization, SIM plus HAM decreased these osteogenic effects (Figure 2). This finding is very interesting, but the molecular mechanism may be complicated. We proposed that the HAM-reduced osteogenic effect of SIM on ADSCs may be due to interference with RUNX-2-regulated expression of ALP and OC and subsequent mineralization.

BMP-2 is known to modulate osteoblastic differentiation through the canonical BMP/Smad pathway and noncanonical BMP pathways [63]. Binding of BMP-2 to the BMP receptor (BMPR) induces Smad phosphorylation and phosphorylated Smad proteins that translocate from the cytoplasm to the nucleus and regulate RUNX-2 expression [63]. On the other hand, noncanonical pathways, such as the MAPK pathway, also play an important role [64]. In the MAPK pathway, JNK and p38 have been shown to regulate OC and ALP expression during BMP-2-induced osteogenesis [65]. Moreover, JNK and p38 are important in regulating RUNX-2 transcriptional activity during osteogenesis [64]. In this study, we found that the SIM group had increased phosphorylated JNK and p38 levels in ADSCs, while these levels were decreased in both the HAM and SIM plus HAM groups (Figure 3D). From this result, we suggest that the suppressive effect of HAM on SIM-induced osteogenesis may be due to suppression of the levels and activation of JNK and p38 and subsequent interference with RUNX-2-regulated osteogenic gene expression.

SOX-9 and RUNX-2 are the principal transcription factors required for chondrogenesis and osteogenesis, respectively [5,66]. BMP-2 promotes SOX-9 expression during chondrogenesis and promotes RUNX-2 expression during osteogenesis [5,62,67,68,69]. In this study, we found that SIM plus HAM enhanced not only BMP-2 but also SOX-9 and RUNX-2 expression in ADSCs (Figure 3). Moreover, CD44 is a main receptor for HA [70]. Blocking the HA-CD44 interaction with IM7 not only significantly reduced SIM-induced BMP-2, SOX-9 and RUNX-2 expression but also decreased the synergistic effect of SIM plus HAM on the expression of these genes in ADSCs (Figure 3). We previously showed that the HA-CD44 interaction in a HAM is important in mediating the chondrogenesis of ADSCs and is mainly mediated through the CD44/ERK/SOX-9 pathway [29,32]. Together with the results of previous studies and these studies, we suggest that the synergistic effect of SIM plus HAM on enhancing BMP-2 expression may contribute to the increased expression of SOX-9 and RUNX-2 in ADSCs. This effect may be through HA-CD44 signaling. Whether the signaling mechanism involves the MAPK pathway needs more investigation in the future.

The most important challenge in ADSC-based articular cartilage tissue engineering is the induction of ADSC chondrogenesis and eventual regeneration of the damaged articular cartilage [9,10,71,72]. We have shown that a 3D HAM (3D ADSCs/HA/Fibrin construct) can enhance the chondrogenesis and neoformation of cartilaginous tissue of ADSCs than ADSCs/Fibrin construct in vitro [29,31]. To clarify whether SIM plus 3D HAM can contribute to neoformation of cartilaginous tissue, we further evaluated the effect of SIM plus 3D HAM in our previously developed 3D ADSCs/HA/Fibrin construct. A hyaline cartilaginous matrix composed mainly of sGAG and collagen type II [2]. SIM treatment further enhanced the production of the normal functional ECM components of articular cartilage, sGAG and collagen type II, in the 3D ADSCs/HA/Fibrin construct in vitro (Figure 1D). To evaluate articular cartilage regeneration, a previously developed model of chondral defects generated in osteochondral core explants ex vivo was used [31]. We have previously reported that the 3D ADSCs/HA/Fibrin construct can regenerate the chondral defect in this model after 4 weeks [31]. Here, we compare whether SIM plus the HA/Fibrin/ADSC construct further enhances the cartilage neoformation of cartilaginous tissue after 4 weeks. In this model, SIM treatment enhanced the neoformation of cartilaginous tissue from the HAM/ADSC construct at the defect site (Figure 4A,B). Collectively, these results suggest that SIM plus 3D HAM (i.e., the HA/Fibrin/ADSC construct) can augment the repair of chondral defects by enhancing neoformation of cartilaginous tissue.

The limitation of this study is that a future study in an animal model of articular cartilage defects for long-term study is needed. Although we found that SIM plus HAM on ADSCs is beneficial for neoformation of cartilaginous tissue and promotes cartilage regeneration in the ex vivo chondral defect model after 4 weeks, the long-term efficacy of SIM plus HAM on articular cartilage regeneration from ADSCs was not demonstrated in vivo. Implantation of the HA/Fibrin/ADSC construct alone or HA/Fibrin/ADSC construct supplemented with SIM should be performed in a large animal that has a thicker articular cartilage. The synthesis of neoformed hyaline cartilage and prevention of undesired bone formation of SIM plus HAM on ADSCs should be also confirmed in vivo. Instead, in this study, we evaluated chondral defect regeneration in a previously established ex vivo chondral defect model [31]. In the future, more suitable criteria should be used to evaluate the success of SIM, HAM and ADSC treatment in the joint cavity environment. The International Cartilage Repair Society (ICRS) II score has been developed for evaluating articular cartilage repair in vivo [71]. The ICRS II score may be used in future studies to evaluate long-term in vivo cartilage repair by SIM, HAM and ADSCs.

## 5. Conclusions

In conclusion, SIM augments the chondrogenic effect of HAMs but does not exert an osteogenic effect on ADSCs. This phenomenon was demonstrated in cultured ADSCs, a 3D HAM (HA/Fibrin/ADSC construct) and an ex vivo chondral defect model. These findings provide new information regarding the local cues by which HA substrates control SIM-induced cell commitment and may enhance the development of chondroinductive factors for articular cartilage regeneration.

## Figures and Tables

**Table 1:** Primer sequences and cycling conditions for real-time PCR.

Gene	PCR Primers Sequence (Forward and Reverse)	Annealing Temperature, °C
Bone Morphogenetic Protein-2 (BMP-2)	Forward: 5′-GGA ATG ACT GGA TTG TGG CT-3′	64
	Reverse: 5′-TGA GTT CTG TCG GGA CAC AG-3′	
SOX-9	Forward: 5′- CTT CCG CGA CGT GGA CAT-3′	55
	Reverse: 5′- GTT GGG CGG CAG GTA CTG-3′	
Collagen Type II	Forward: 5′-CAA CAC TGC CAA CGT CCA GAT-3′	61
	Reverse: 5′-TCT TGC AGT GGT AGG TGA TGT TCT-3′	
Aggrecan	Forward: 5′-ACA GCT GGG GAC ATT AGT GG-3′	61
	Reverse: 5′-GTG GAA TGC AGA GGT GGT TT-3′	
Runt-Related Transcription Factor 2 (RUNX-2)	Forward: 5′-ACA GCT GGG GAC ATT AGT GG-3′	58
	Reverse: 5′-GTG GAA TGC AGA GGT GGT TT-3′	
Osteocalcin (OC)	Forward: 5′- GTG CAG AGT CCA GCA AAG GT-3′	61
	Reverse: 5′- CGA TAG GCC TCC TGA AAG C-3′	
Alkaline Phosphatase (ALP)	Forward: 5′- CCT CCT CGG AAG ACA CTC TG -3′	61
	Reverse: 5′- GCA GTG AAG GGC TTC TTG TC -3′	
Glyceraldehyde-3-phosphate-dehydrogenase	Forward: 5′-TCT CCT CTG ACT TCA ACA GCG AC-3′	61
(GAPDH)	Reverse: 5′-CCC TGT TGC TGT AGC CAA ATT C-3′	
Cycling Conditions	Denature: 95 °C for 30 s, 95 °C for 4 min, followed by 35 cycles of 95 °C for 10 s, 55–61 °C (shown in column of Annealing Temperature) for 15 s and 72 °C for 15 s	

## Data Availability

Data available on request.
